# Concurrent Repetitions Overestimate Hamstring:Quadriceps Ratios at Extended Knee Joint Positions: Implications for Clinical Practice

**DOI:** 10.1111/sms.70049

**Published:** 2025-04-04

**Authors:** Gareth Nicholson, Josh Walker, Chris Brogden, Tobias Alt

**Affiliations:** ^1^ Carnegie School of Sport Leeds Beckett University Leeds UK; ^2^ School of Health Leeds Beckett University Leeds UK; ^3^ Department of Biomechanics, Performance Analysis, and Strength & Conditioning Olympic Training and Testing Centre Dortmund Germany

**Keywords:** hamstring, isokinetic dynamometry, kinematics, measurement error, muscle imbalance

## Abstract

Most measurements of isokinetic hamstring:quadriceps (H:Q) strength ratios are conducted using concurrent repetitions, whereby active knee extension is immediately followed by active knee flexion. To reduce the influence of the stretch‐shortening cycle and limit axis misalignment, isolated repetitions have been recommended, whereby extension and flexion are completed separately. To inform screening protocols, this study examined the effect of concurrent and isolated trials on discrete and angle‐specific H:Q ratios. Fifteen males (age: 27 ± 4 years; height: 184 ± 9 cm; body mass: 80 ± 9 kg) performed isokinetic tests of the knee flexors and extensors (60°/s) using concurrent and isolated trials while sagittal kinematics were captured (100 Hz). Statistical parametric mapping enabled the effects of protocol type (concurrent vs. isolated) and axis misalignment (uncorrected vs. corrected) to be compared. Uncorrected data resulted in an underestimation of discrete conventional (−10.17%, *p* < 0.001) and functional (−9.21%, *p* <  0.05) ratios, with differences being observed for all angle‐specific ratios (*p* < 0.001). The use of concurrent repetitions resulted in a significant overestimation of the conventional H:Q ratio (+7.41%, *p* < 0.05) with the differences being most prevalent at more extended (24°–45° knee flexion, *p* < 0.05) knee joint positions. Dynamometer users should be aware that concurrent repetitions increase the likelihood of “false‐negative” injury risk categorization. Nevertheless, the common practice of using uncorrected data from concurrent repetitions does not lead to significant differences in discrete or angle‐specific H:Q ratios when compared with corrected data obtained from isolated repetitions.

## 1 Introduction

Isokinetic dynamometry is considered the best method available to assess single‐joint mechanical function [1]. The method measures joint moments through the full range of movement at a predetermined angular velocity. Joint moment measurements using isokinetic dynamometry typically assess strength attributes through variables such as peak joint moment and angle of peak joint moment [2]. Routine assessments of the knee extensors and flexors are commonplace, with the joint moment ratio between the hamstrings and quadriceps (known as the H:Q ratio) assessing athletes' strength balance around the knee joint. Strength imbalances between the hamstrings and quadriceps have previously been associated with hamstring and knee injuries [3]. As a result, assessments of the H:Q ratios are routinely used as markers of recovery in return to play (RTP) protocols [4] and as screening tools when identifying injury risk [5].

Typically, H:Q ratios are obtained using the peak joint moment of the flexors and extensors [6]. Despite the common use of these discrete ratios in isokinetic screening protocols [7], they provide only a single value based on peak joint moments. These individual moments occur at different joint angles [8] and do not make use of the fact that isokinetic assessments provide range of motion (ROM)‐specific information regarding knee joint function [9]. It is therefore not surprising that a number of studies have recommended a time‐series metric generated from angle‐specific joint moments [10, 11, 12, 13, 14]. A recent study by Lunn et al. [14] compared discrete and angle‐specific ratios in Premier League soccer players and demonstrated that angle‐specific ratios more closely reflect the instantaneous relationship between knee flexor and extensor moments at different joint positions. Although longitudinal research is needed to better understand the predictive capacity of angle‐specific ratios in anterior cruciate ligament (ACL) [10] and hamstring [15] injury screening protocols, it is clear that H:Q ratios based on peak joint moments alone oversimplify knee joint strength imbalances, potentially leading to incorrect return‐to‐sport decisions [16]. As such, research suggests that where possible, angle‐specific (or time‐series) analyses of H:Q strength should be conducted to accurately understand muscle strength balance through the ROM [14].

Most testing of isokinetic H:Q ratios is conducted in a seated position and involves concurrent repetitions (CONC), where the active extension of the knee joint is immediately followed by active flexion to return the joint to its starting position [17, 18, 19]. A different approach is to conduct assessments using isolated repetitions (ISOL) in a single direction (i.e., extension or flexion), whereby the return to the starting position occurs in a relaxed muscular state. While this approach may extend the time required for data collection compared to CONC protocols (two separate tests instead of one), the use of ISOL repetitions has been advocated to improve selective muscle activation [20, 21]. Furthermore, ISOL might also reduce the influence of the stretch‐shortening cycle because the antagonistic muscle groups are required to move quickly into the role of agonist during the transition from knee extension to flexion (and vice versa) for protocols that involve CONC repetitions. Although the utilization of CONC would be more “time‐efficient,” these protocols might influence the measured H:Q ratios and subsequent injury risk categorization. Despite this, research to date has not compared the effects of CONC and ISOL protocols on time‐series analyses and angle‐specific H:Q ratios, which is essential information for the implementation of such analyses moving forward.

Despite the use of positioning and stabilization procedures that align with the manufacturer's recommendations, a number of studies have reported knee extensor and flexor peak moments are also affected by the misalignment of the rotation axes between the knee joint and the dynamometer during repetitions [22, 23, 24, 25, 26]. Furthermore, the magnitude of misalignment and resulting error is known to vary through the ROM [25], which likely affects angle‐specific H:Q ratios and subsequent interpretation by the clinicians. While some level of axis misalignment will also exist during ISOL repetitions [26], practitioners can be confident that the influence from preceding, active muscular contractions will be limited, like those experienced during CONC testing protocols. Although the effects of axis misalignment during CONC repetitions may be different from those experienced during ISOL repetitions, a poor understanding currently exists regarding the magnitude and direction of any error introduced by axis misalignment during CONC and ISOL protocols [26]. As such, the influence that “time‐efficient” CONC protocols have on angle‐specific H:Q ratios and subsequent risk categorization remains unclear.

Research that compares kinetic and kinematic responses to concurrent and isolated isokinetic protocols is needed to enable practitioners to correctly implement and understand angle‐specific H:Q analyses. From a practical perspective, this establishes whether angle‐specific analyses should continue using an approach that facilitates “time‐efficient” data collection or use a slower approach that provides more accurate, reliable, and athlete‐specific data. This information is essential for practitioners and scientists who implement isokinetic tests for screening and/or RTP purposes to inform the decision of isokinetic protocols and to ensure the accurate interpretation of diagnostic data. As such, the aim of this study was to examine the effects of protocol type (concurrent versus isolated trials) and axis misalignment (corrected versus uncorrected) on the isokinetic H:Q ratios (discrete and angle‐specific) obtained from seated knee flexion and extension testing at 60°/s.

## 2 Materials and Methods

### 2.1 Participants

Fifteen healthy male participants (age: 27 ± 4 years; height: 184 ± 9 cm; body mass: 80 ± 9 kg) volunteered to participate in this study. Participants were recruited from a range of sporting backgrounds (track and field, bobsleigh, and soccer), were experienced with resistance training, and were free from hamstring strains or knee injuries in the 2 years preceding data collection. This study gained ethical approval from the local university ethics committee, and testing was carried out in accordance with the Declaration of Helsinki.

### 2.2 Procedures

All participants completed three sessions that included one familiarization session and two testing sessions, separated by at least 48 h. Strength testing of the knee joint on the left limb was conducted using an isokinetic dynamometer (IsoMed 2000, D&R Ferstl GmbH, Hemau, Germany). To conduct testing, participants were seated on the dynamometer with the involved limb distally fixed with a Velcro strap [23, 24] while the participants contracted their knee extensors at ~50% intensity. A hand scale (First Australia, Timetron, Vienna, Austria) maintained a standardized strap traction of 120 N, with a tighter fastening (200 N) having previously been shown to significantly reduce peak moments [27]. Tests were executed in a seated position with the hip joint placed at ~60° flexion. An experienced operator aligned the dynamometer axis with the participants' lateral femoral epicondyle (using a laser pointer) while in a preactivated muscular state at 0° knee flexion [28]. Finally, a double shin pad was placed 2–3 cm proximal to the medial malleolus [29, 30]. Once in a fixed position, a gravity correction was applied to the dynamometer's joint moment signal, and the ROM was set at 90°, reaching full knee extension (=0°).

Two high‐speed cameras (DR1‐DR2048‐192‐G2, Photonfocus, Lachen, Switzerland) per body side captured the isokinetic movements at 100 Hz (TEMPLO 2019.1.578, Contemplas, Kempten, Germany). Additional lighting (F02T75, ALT, Taipei City, Taiwan) improved the retroreflective markers' visibility which were attached to the participant's body at the sixth rib, greater trochanter, lateral femoral epicondyle, and the dynamometer's lever arm. The testing environment was calibrated by a calibration frame (158 × 60 × 107 cm), and a calibration star (40 × 40 × 40 cm) was mounted to the dynamometer's rotation axis prior to the tests to determine its spatial orientation. Kinematic analyses were executed with VICON Peak Motus (V10.0.1, New York/NY/USA).

At the start of each testing session, an isokinetic warm‐up consisting of 15 submaximal (~60%–80% effort) concentric and eccentric repetitions of the extensors and flexors was undertaken at an angular velocity of 60°/s, as is commonplace during H:Q screening protocols [7, 28]. Isokinetic testing of the knee flexors and extensors was then conducted at 60°/s, with the testing velocity aligning with the conditions commonly adopted during isokinetic knee joint protocols [7, 18]. Trials were conducted in a concurrent manner (CONC), whereby concentric knee extension was immediately followed by concentric knee flexion to return to the start position. Eccentric knee flexion trials were concurrently conducted with concentric knee flexion trials. To reduce the excessive accumulation of muscular fatigue, CONC tests were always separated by one sequence of idle movements [26]. Trials were also conducted in an isolated manner (ISOL) in a single direction (either concentric knee extension, concentric knee flexion, or eccentric knee flexion) whereby the return to the starting position occurred in a relaxed muscular state [20, 21]. For the CONC and ISOL protocols, participants were permitted five repetitions (two submaximal and three maximal‐effort repetitions) where participants were verbally encouraged to promote maximal exertion throughout the full ROM [21]. Sets were separated by 180‐s rest intervals. Signals for joint moment and joint position were recorded by the dynamometer's software at 200 Hz and synchronized with the camera‐based data acquisition (NI USB6210, National Instruments, Austin/TX, USA).

### 2.3 Data Processing

All data processing was conducted in MATLAB (R2022a, MathWorks Inc., USA). Imported joint moment and positional data were initially filtered using a recursive, second‐order, low‐pass Butterworth filter with a cut‐off frequency of 10 Hz [13]. From the three maximal‐effort repetitions in each testing condition, the “best trial” (determined using peak joint moment) was used for analysis. Peak joint moment was defined as the maximum joint moment achieved throughout the analyzed range (for corrected and uncorrected data), which was between 20° and 80° of knee flexion. This range was selected to avoid any participants being outside the isokinetic range (i.e., less than 95% of the target angular velocity [1, 28]) and to minimize any effects of antagonistic muscle–tendon unit stiffness on the measured joint moment. Knee joint angular velocity was calculated as the first time‐derivative of joint angle.

Conventional H:Q ratio was calculated as the ratio between peak joint moment in the knee flexors and extensors at 60°/s. Functional H:Q ratio was calculated as the ratio between peak joint moment in the flexors during the eccentric condition at 60°/s and the concentric extensors at 60°/s. In addition to these discrete ratios, angle‐specific H:Q ratios (conventional and functional, defined in the same way as above) were computed as the ratio between flexor and extensor joint moments for all joint angles where joint moment and angle data were analyzed (between 20° and 80° of knee flexion).

For the kinematic analysis, the retroreflective marker pathways were tracked to obtain sagittal plane kinematics in line with previous research [27, 31]. The axes and movement planes were defined according to the human body (*x*: anteroposterior; *z*: transverse). The corrected (i.e., for axis misalignment) knee moments at the knee joint were calculated as previously described: [32]

$$M_{\mathrm{CB}}=M_{\mathrm{DB}}\cdot d_{\mathrm{CB}}/d_{\mathrm{DB}}$$

where *M* = moment, *d* = lever arm, CB = camera‐based, and DB = dynamometer‐based.

### 2.4 Statistical Analysis

All statistical analyses were conducted in SPSS (version 28, IBM, USA) and MATLAB. Two‐way analyses of variance (ANOVA) with repeated measures were used to compare the main effects of protocol type (i.e., CONC versus ISOL) and axis misalignment (uncorrected versus corrected), as well as the interaction between main effects. These tests were conducted for peak joint moments and discrete H:Q ratios. Additionally, angle‐specific H:Q ratios were compared with statistical parametric mapping (SPM) using the open‐source “spm1d” MATLAB package (version M.0.4.10) [33]. This analysis compared main effects of protocol type and axis misalignment, as well as the interaction effects, across the full analyzed ROM. Comparisons between discrete data were accompanied with partial eta‐squared (*η*_*p*_^*2*^), which were interpreted as: small = 0.01, medium = 0.06, and large = 0.14 [34]. Significance levels were set at *p* < 0.05.

## 3 Results

Descriptive statistics for peak join moments can be seen in Table 1. Significant main effects of axis misalignment were observed for concentric knee extensor (uncorrected: 250.99 ± 52.15 N m, corrected: 235.69 ± 45.39 N m; *p <* 0.001), concentric knee flexor (uncorrected: 131.00 ± 20.61 N m, corrected: 137.22 ± 21.53 N m; *p <* 0.001), and eccentric knee flexor (uncorrected: 169.83 ± 32.10 N m, corrected: 175.60 ± 31.24 N m; *p* = 0.010) peak moment. For the eccentric knee flexor peak moment, a significant main effect was also reported for the protocol type, with higher values reported for the isolated trials (concurrent: 168.04 ± 31.61 N m, isolated: 177.38 ± 31.29 N m; *p* = 0.014). No significant interactions were observed for any of the peak joint moment variables.

**TABLE 1:** Mean ± SD and ANOVA results for peak joint moment during the different testing conditions.

		CONC (mean ± SD)	ISOL (mean ± SD)	ANOVA
Concentric knee extensors (N m)	Uncorrected Corrected	248 ± 53 233 ± 46	254 ± 53 238 ± 46	Uncorrected versus corrected: *F* = 20.86, *p* < 0.001, *η*_*p*_^2^ = 0.598 CONC versus ISOL: *F* = 3.14, *p* = 0.098, *η*_*p*_^2^ = 0.183 Interaction: *F* = 1.23, *p* = 0.286, *η*_*p*_^2^ = 0.081
Concentric knee flexors (N m)	Uncorrected Corrected	134 ± 21 140 ± 22	128 ± 20 134 ± 21	Uncorrected versus corrected: *F* = 48.10, *p* < 0.001, *η*_*p*_^2^ = 0.775 CONC versus ISOL: *F* = 3.14, *p* = 0.082, *η*_*p*_^2^ = 0.200 Interaction: *F* < 0.01, *p* = 0.961, *η*_*p*_^2^ < 0.001
Eccentric knee flexors (N m)	Uncorrected Corrected	166 ± 33 170 ± 31	174 ± 32 181 ± 31	Uncorrected versus corrected: *F* = 9.79, *p* = 0.010, *η*_*p*_^2^ = 0.386 CONC versus ISOL: *F* = 7.93, *p* = 0.014, *η*_*p*_^2^ = 0.361 Interaction: *F* = 1.74, *p* = 0.208, *η*_*p*_^2^ = 0.111

*Note:* Uncorrected joint moments denote the joint moment data provided by the isokinetic dynamometer, while corrected moments account for changes in the moment arm (see Section 2).

Abbreviations: CONC, concurrent extension/flexion actions; ISOL, isolated extension/flexion actions.

When discrete H:Q ratios were computed, significant main effects of axis misalignment were observed for the conventional (uncorrected: 0.53 ± 0.09 N m, corrected: 0.59 ± 0.09 N m, *p <* 0.001) and functional (uncorrected; 0.69 ± 0.14 N m, corrected: 0.76 ± 0.15 N m, *p* = 0.010) ratios (Table 2) indicating significantly greater H:Q ratios (conventional and functional) when axis misalignment is accounted for. For the discrete conventional strength ratio, a significant main effect was also reported for the protocol type with higher values reported for the concurrent trials (concurrent: 0.58 ± 0.09 N m, isolated: 0.54 ± 0.10 N m, *p* = 0.036). No significant interactions were observed for any of the discrete H:Q ratios. The discrete H:Q ratios (conventional and functional) for each participant are reported in Supporting Information (Data S1 and S2) which support the main effects of axis alignment and protocol type described above.

**TABLE 2:** Mean ± SD and ANOVA results for H:Q ratios (based on peak joint moments) during the different testing conditions.

		CONC (mean ± SD)	ISOL (mean ± SD)	ANOVA
Conventional H:Q ratio	Uncorrected Corrected	0.55 ± 0.08 0.61 ± 0.09	0.51 ± 0.09 0.57 ± 0.09	Uncorrected versus corrected: *F* = 130.79, *p* < 0.001, *η*_*p*_^2^ = 0.903 CONC versus ISO: *F* = 5.40, *p* = 0.036, *η*_*p*_^2^ = 0.278 Interaction: *F* = 0.07, *p* = 0.803, *η*_*p*_^2^ = 0.005
Functional H:Q ratio	Uncorrected Corrected	0.68 ± 0.14 0.75 ± 0.15	0.70 ± 0.15 0.78 ± 0.16	Uncorrected versus corrected: *F* = 55.16, *p* = 0.010, *η*_*p*_^2^ = 0.798 CONC versus ISOL: *F* = 1.21, *p* = 0.291, *η*_*p*_^2^ = 0.079 Interaction: *F* = 4.28, *p* = 0.058, *η*_*p*_^2^ = 0.234

*Note:* Uncorrected joint moments denote the joint moment data provided by the isokinetic dynamometer, while corrected moments account for changes in the moment arm (see Section 2).

Abbreviations: CONC, concurrent extension/flexion actions; ISOL, isolated extension/flexion actions.

**FIGURE 1:**
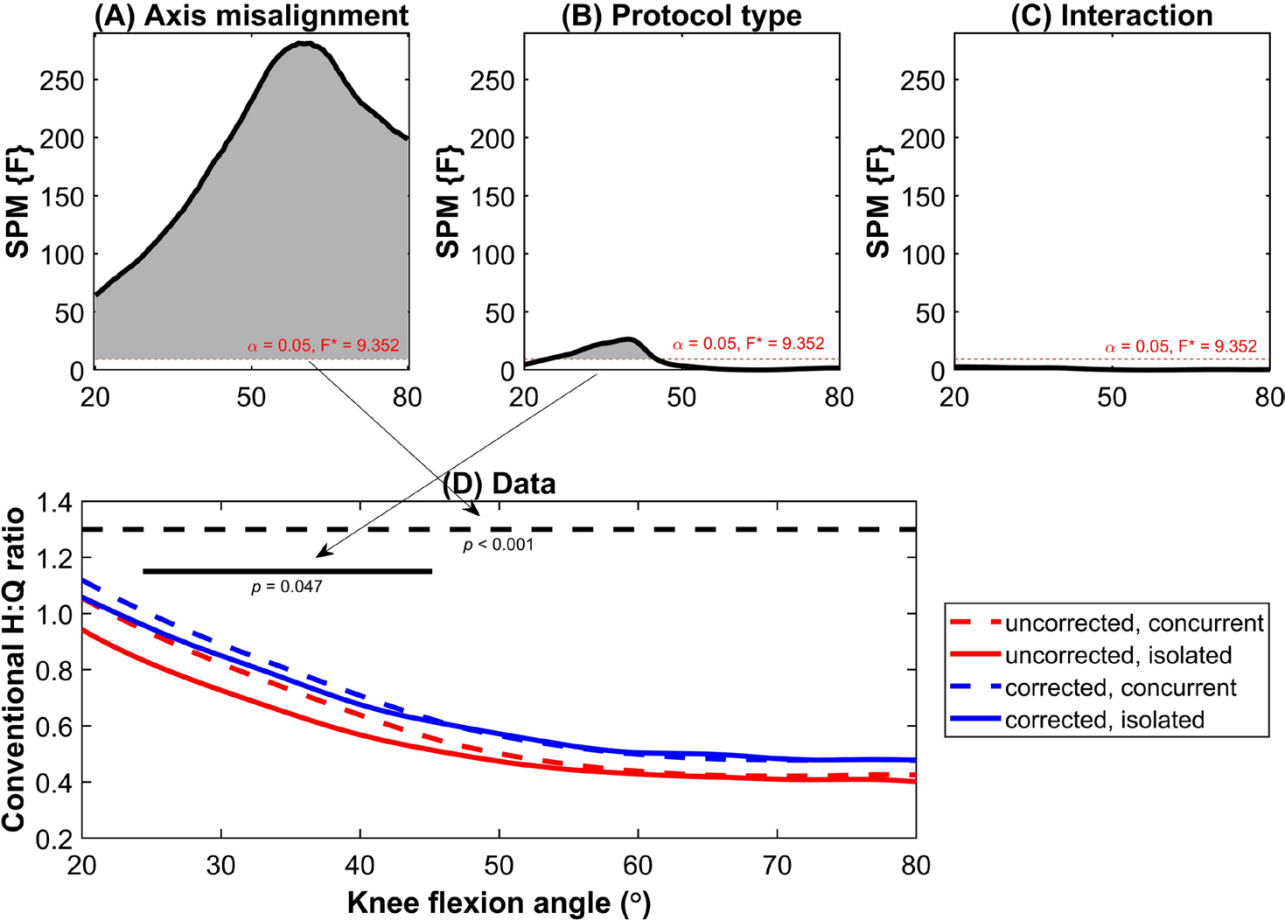
Output of the statistical parametric mapping (SPM) for the conventional H:Q ratios showing the main effects of: (A) axis misalignment (i.e., uncorrected versus corrected), (B) protocol type (i.e., concurrent versus isolated), and (C) the interaction between these factors. (D) Angle‐specific conventional strength ratio presented as a function of joint angle with the associated SPM output.

When conventional strength ratio was analyzed as a function of joint angle (Figure 1), a significant main effect of axis misalignment (uncorrected: 0.57 ± 0.17, corrected: 0.63 ± 0.18, *p <* 0.001) was observed throughout the entire analyzed ROM (20°–80° of knee flexion) with higher values being reported when axis misalignment is accounted for. A significant main effect was also reported for the protocol type (concurrent: 0.62 ± 0.19, isolated: 0.57 ± 0.16, *p* = 0.047), but the greater values for concurrent trials were confined to more extended knee joint positions (from 24° to 45° of knee flexion). No significant interaction was observed at any point within the analyzed ROM.

**FIGURE 2:**
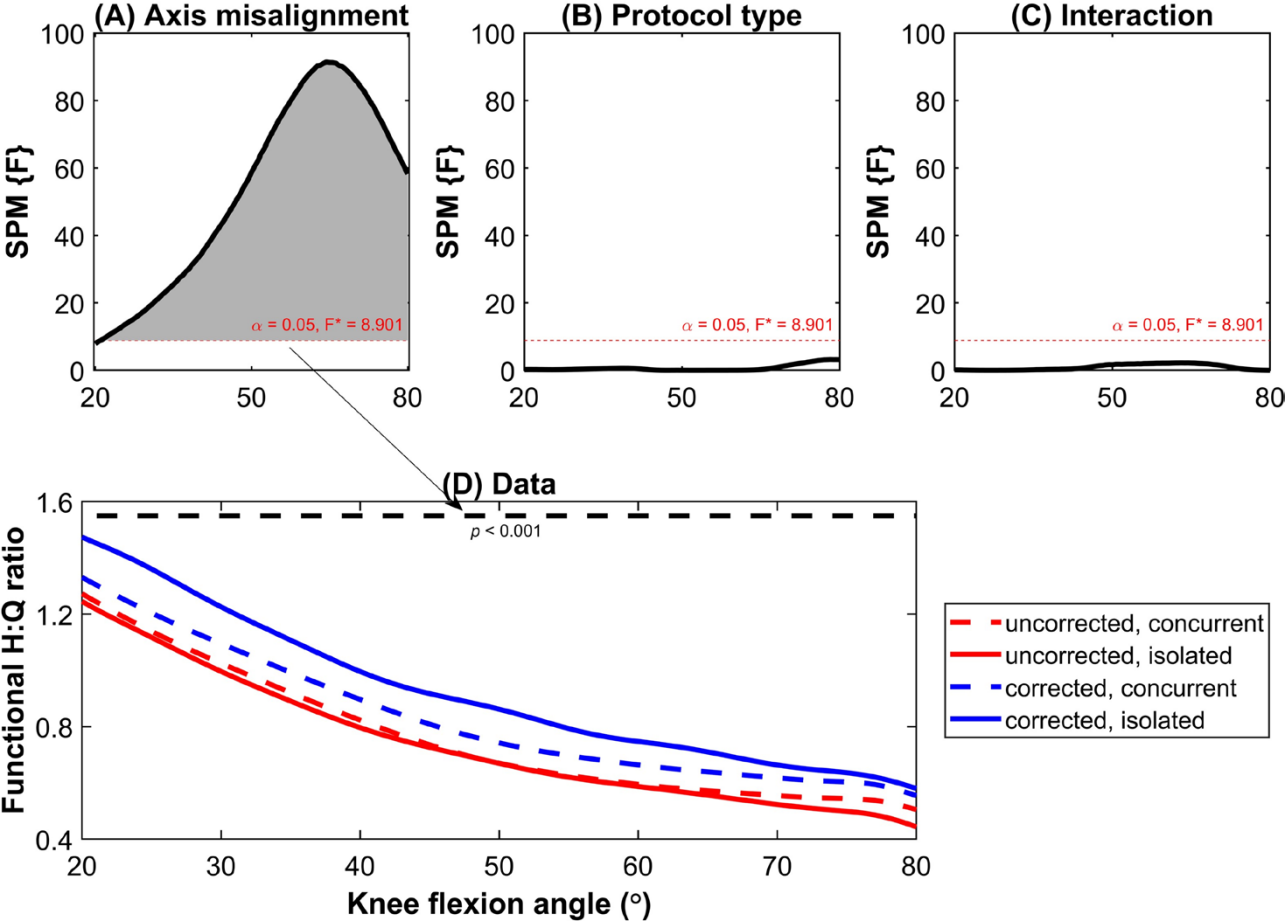
Output of the statistical parametric mapping (SPM) for the functional H:Q ratios showing the main effects of: (A) axis misalignment (i.e., uncorrected versus corrected), (B) protocol type (i.e., concurrent versus isolated), and (C) the interaction between these factors. (D) Angle‐specific functional strength ratio presented as a function of joint angle with the associated SPM output.

When functional strength ratio was analyzed as a function of joint angle (Figure 2), a significant main effect of axis misalignment (uncorrected: 0.75 ± 0.22, corrected: 0.82 ± 0.22, *p <* 0.001) was observed throughout the entire analyzed ROM (20° and 80° of knee flexion) with higher values being reported when axis misalignment is accounted for. In contrast to the conventional ratio, no significant main effect was reported for the protocol type (concurrent: 0.79 ± 0.22, isolated: 0.77 ± 0.22) with significant interactions between the two factors also being absent.

## 4 Discussion

The aim of this study was to examine the effects of protocol type (CONC versus ISOL) and axis misalignment (corrected versus uncorrected) on the H:Q ratios obtained from seated isokinetic tests at 60°/s. The findings demonstrate that the use of isolated repetitions had a significant effect on the conventional H:Q ratio, with the differences being most prevalent at more extended knee joint positions (24° to 45° knee flexion). Axis misalignment also had a significant effect on discrete and angle‐specific H:Q ratios, with differences being evident throughout the entire ROM for both conventional and functional ratios. Despite the independent effects of axis misalignment and repetition type, no differences were observed when corrected, and isolated repetitions were compared to uncorrected, concurrent repetitions. These findings have important implications for practitioners during the design and analysis of testing protocols aimed at assessing angle‐specific H:Q ratios.

The significant effect that repetition type (i.e., concurrent versus isolated) had on the conventional H:Q ratio outlines an important consideration when planning isokinetic strength assessments. The use of concurrent repetitions resulted in a significant underestimation of eccentric peak knee flexor moment (−5.27%) and a significant overestimation of the conventional H:Q ratio (+7.41%) when compared to isolated repetitions. Furthermore, the fact that the differences in the conventional H:Q ratio were more pronounced at more extended knee joint angles (close to the angle of peak flexor moment) means that the type of repetitions administered may be particularly important during angle‐specific analyses. It has been suggested that the knee moment data captured during isolated repetitions may be impacted by an improved ability to selectively activate the knee extensors and flexors [20, 21]. In addition, the influence of the stretch‐shortening cycle could also be suggested to be reduced because the passive preparation to each voluntary contraction likely influences the muscle–tendon length and activation status of the agonist at the commencement of each trial. Future research which incorporates objective markers of voluntary activation (i.e., interpolated twitch) and muscle activation (surface electromyography) is required to better understand the mechanisms underlying the effect of repetition type. Nevertheless, the elevated conventional H:Q ratio in concurrent repetitions carries particular implications given the prevalence of this parameter in RTP protocols [35, 36]. Specifically, the common utilization of “time‐efficient” CONC protocols suggests players could be regarded as being “well balanced” when in fact they have relatively weak hamstrings at more extended joint positions, which would be a greater concern for injury risk [37]. Therefore, where there is limited time available for testing, practitioners should be mindful of potential “false‐negative” findings in individuals who are close to thresholds used for injury risk categorization.

The finding that axis misalignment had a significant effect on the data obtained from the isokinetic testing protocols is consistent with previous research which investigated isokinetic ankle [32] and knee [27, 28, 31] protocols, and occurred despite the use of positioning and stabilization procedures that align with the current recommendations. The uncorrected data (ISOL and CONC combined) overestimated concentric knee extensor moment (+6.49%) and underestimated eccentric knee flexor (−3.29%) and concentric knee flexor (−4.53%) moments when compared to the data that were corrected for axis misalignment. This manifested as an underestimation of the conventional (−10.17%) and functional (−9.21%) H:Q ratios when computed from peak joint moments. Importantly, the present data show that the underestimation caused by axis misalignment is present throughout the analyzed ROM, which is particularly important considering the increasing utilization of angle‐specific H:Q ratios [10, 11, 12, 13, 14]. Practitioners should therefore be mindful that errors caused by axis misalignment influence angle‐specific H:Q ratios in a similar way to their discrete alternatives. While the consistent underestimation presents some challenges in the use of H:Q ratios in the accurate interpretation of hamstring or ACL injury risk, the underestimation means that the uncorrected data are more likely to highlight a player as being “at risk” of hamstring injury when in fact they are not (i.e., “false‐positive”). It therefore appears that the common use of uncorrected knee moment data (likely due to equipment constraints) to compute discrete and angle‐specific H:Q ratios provides a more cautious estimation of H:Q ratios which is preferable to a “false‐negative” scenario.

The merits of isokinetic testing as an injury screening tool are often justified based on historical studies [38] that have utilized discrete H:Q ratios calculated from concurrent repetitions without correction for axis misalignment. The need for longitudinal research which examines the predictive capacity of angle‐specific ratios in ACL and hamstring injury screening protocols has recently been highlighted [15]. The present findings, however, highlight the need to also consider axis misalignment correction and repetition type in exploring the association between injury and angle‐specific H:Q ratios. Based on the present findings, the utilization of knee moment data which are corrected for axis misalignment (i.e., camera‐based analyses) is the preferred option for computing discrete and angle‐specific H:Q ratios. Nevertheless, such an approach might not always be viable because of additional equipment requirements and time restrictions. If uncorrected knee moment data are to be used, practitioners should be mindful that uncorrected knee extensor and flexor moments will always contain measurement error as the knee joint does not maintain a fixed axis of rotation [24, 25]. Nevertheless, it is important to explore steps that may help to minimize axis misalignment errors or at least to understand the data in a way which helps to draw the correct interpretation from uncorrected analyses. Despite the differing magnitudes of error that are caused by axis misalignment for peak extensor and flexor moments [25], the use of isolated repetitions in the present study did not minimize these errors. This is based on the absence of a significant interaction between repetition type and axis misalignment, meaning that the use of both isolated repetitions and corrected joint moment data does not result in more accurate H:Q ratios when compared with uncorrected data obtained from concurrent repetitions. Practitioners can therefore make decisions regarding repetition type (concurrent versus isolated) and moment calculation (correct versus uncorrected) independently.

When interpreting the findings of this study, practitioners should be mindful of some limitations. There is an absence of an objective marker (e.g., interpolated twitch technique) to permit the confirmation of full voluntary activation during the isokinetic protocols. Only one conventional and one functional strength ratio were computed, despite various iterations being presented across the literature [7]. We chose testing conditions (seated, 60°/s) that were commonly reported in the literature [18, 39] and utilized for athlete screening and RTP protocols. The study was conducted using one commercially available dynamometer; because other manufacturers (e.g., Kin‐Com, Cybex, and Biodex) permit different levels of system and component compliance (e.g., padding), practitioners should be mindful that axis misalignment could vary between laboratories/manufacturers. Lastly, knee flexor and extensor protocols were performed on separate visits to limit the effects of cumulative fatigue. As such, the H:Q ratios computed from isolated repetitions may have been affected by variations in participant readiness on each testing visit.

## 5 Perspective

The findings of this study show that “time‐efficient” isokinetic protocols that utilize uncorrected knee moment data or concurrent repetitions will produce differences in H:Q ratios compared to more time‐consuming protocols (i.e., isolated repetitions) that necessitate additional equipment (i.e., camera‐based analyses). Although their effects appear to be independent of each other, the type of protocol (i.e., concurrent versus isolated) and axis misalignment represent important considerations for practitioners when implementing isokinetic protocols in RTP and injury screening protocols. These considerations warrant consideration during angle‐specific analyses, which are being increasingly utilized to capitalize on the time‐series data available from isokinetic dynamometry. While some of these considerations likely depend on the time constraints and equipment limitations imposed on the medical team, the data demonstrate the implications of the decisions on the accurate interpretation of H:Q ratios and subsequent injury risk categorization.

## Conflicts of Interest

The authors declare no conflicts of interest.

## Supporting information


Data S1.

Data S2.

## Data Availability

The data that support the findings of this study are available from the corresponding author upon reasonable request.
