# Supplementary figures and images for: Concurrent Repetitions Overestimate Hamstring:Quadriceps Ratios at Extended Knee Joint Positions: Implications for Clinical Practice
Source: Scand J Med Sci Sports. 2025 Apr 4;35(4):e70049. doi: 10.1111/sms.70049 (PMC11971590; doi:10.1111/sms.70049)

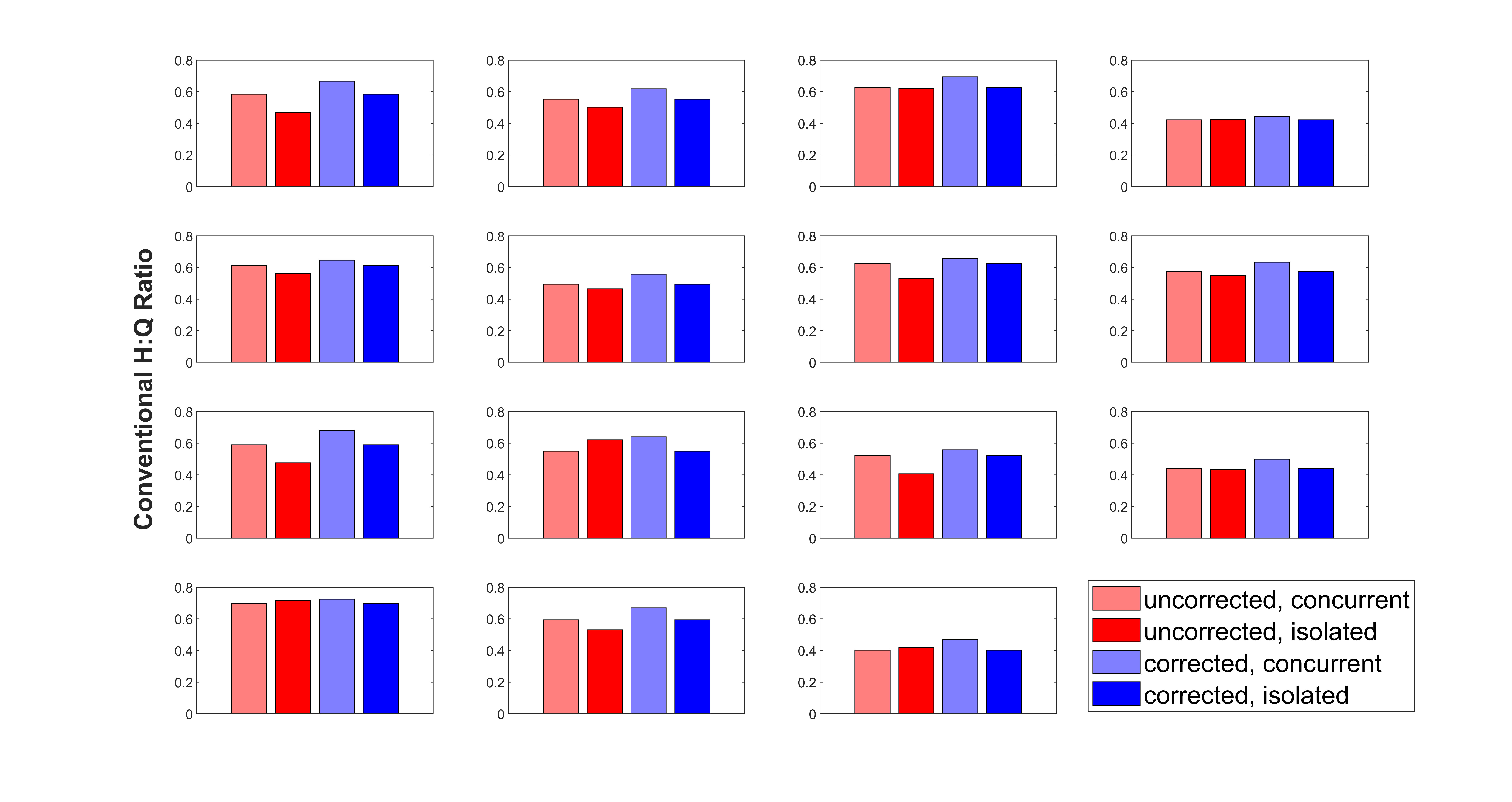

Supplement: Supplementary file 1 — Data S1. [file SMS-35-e70049-s002.tif]

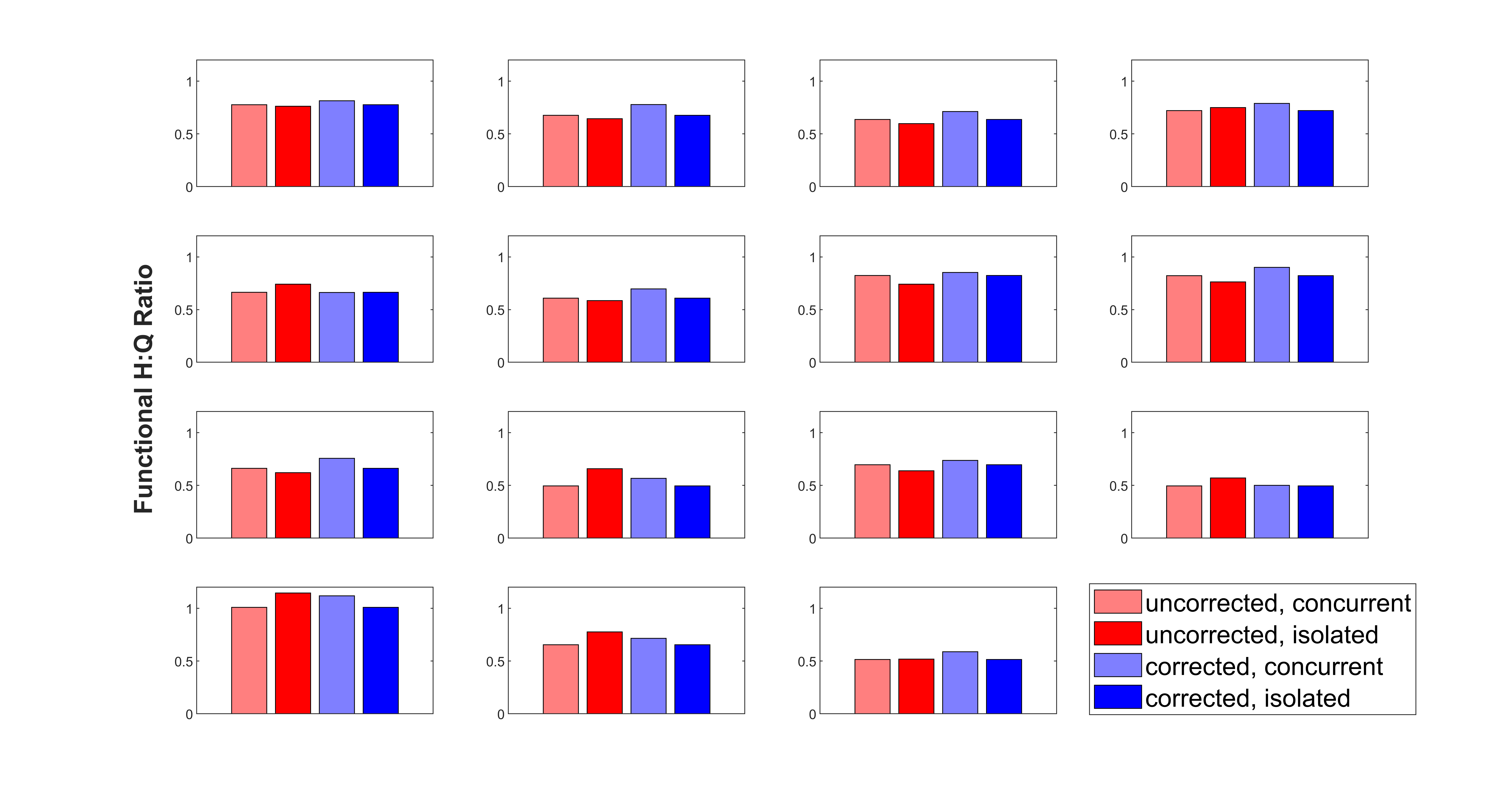

Supplement: Supplementary file 2 — Data S2. [file SMS-35-e70049-s001.tif]
